# Comprehensive Microbiome and Metabolome Analyses Reveal the Medicinal Components of *Paeonia lactiflora*

**DOI:** 10.3390/plants12081612

**Published:** 2023-04-10

**Authors:** Liping Yang, Mengdi Zhou, Mengting Zu, Jiajia Zuo, Yingdan Yuan

**Affiliations:** College of Horticulture and Landscape Architecture, Yangzhou University, Yangzhou 225009, China

**Keywords:** *Paeonia lactiflora*, microbiome, metabolome, ornamental cultivar

## Abstract

*Paeonia lactiflora* Pall. is not only a traditional ornamental plant, but also an important medicinal plant. Currently, some *P. lactiflora* cultivars are used for ornamental purposes, but their potential medicinal value is ignored. To explore the medicinal potential of the ornamental varieties, the medicinal cultivar ‘Hangbaishao’ (HS) and the ornamental cultivar ‘Zifengyu’ (ZFY) were selected, and microbiome and metabolome analyses were performed to compare the composition of the endophytes and metabolites in the roots. The diversity and abundance of bacteria were not significantly different between HS and ZFY; however, the diversity and abundance of endophytic fungi in the ornamental cultivar ZFY were much higher than those in the medicinal cultivar HS. The flavonoids and phenolic acid contents of the ornamental cultivar ZFY were significantly higher than those of the medicinal cultivar HS, indicating that ZFY has medicinal value. The differences in root endophytes between HS and ZFY may lead to differences in phenolic acids and flavonoids. To explore the relationship between endophytes and the accumulation of phenolic acids and flavonoids, a joint analyses of the microbiome and metabolome were performed. The key bacterium, *Ruminococcaceae bacterium* GD7, led to the accumulation of phenolic acids and flavonoids in the ZFY. This study contributes to future research on the potential medicinal value of ornamental *P. lactiflora* and provides a new approach for realizing the ‘dual use of medicine and appreciation’ of *P. lactiflora*.

## 1. Introduction

*Paeonia lactiflora* Pall., a perennial herb in the *Paeoniaceae* family, is native to Eurasia [1]. It is a famous traditional flower in China that has both ornamental and medicinal value [2,3]. The medicinal value of *P. lactiflora* has been studied extensively. Studies have found that *P. lactiflora* has a variety of pharmacological activities, such as anti-inflammatory, analgesic, antibacterial, antioxidant, and anti-cancer activities [4,5,6,7]. As the main medicinal part, the root of *P. lactiflora* contains a variety of medicinal components, including glycosides, terpenoids, flavonoids, volatile oils, phenols and sugars [8,9,10,11,12,13], among which paeoniflorin is the most important bioactive substance [14]. Shibata et al. first extracted paeoniflorin from *P. lactiflora* roots in 1963 [15]. Xin et al. reviewed the mechanism of action of paeoniflorin and its therapeutic potential in inflammatory diseases [16]. Liu et al. demonstrated the antioxidant activity of flavonoids in *P. lactiflora* and showed that they can be used as natural antioxidant active substances [17]. Therefore, improving medicinal ingredients in the roots of *P. lactiflora* is an important scientific issue.

Endophytic microorganisms are microorganisms that inhabit plants and have been proven to be important regulators of root health [18]. Endophytic bacteria promote plant growth by secreting phytohormones, improving nutrition through bidirectional nutrient transfer, and enhancing plant health by protecting plants from pathogens [19]. Lata et al. reviewed and discussed the potential of endophytes to promote plant growth under drought, high temperatures, high salinity, and poor nutrient availability [20]. Endophytes are ubiquitous in plants and have been reported in various hosts. Inoculation of *Oryza Sativa* with endophytic bacteria (*Enterobacter ludwigii* SAK5 and *Exiguobacterium indicum* SA22) can alleviate the stress of cadmium and nickel in *O. Sativa* and promote growth [21]. *Bacillus sp.* LZR216, isolated and identified in the roots of *Arabidopsis thaliana*, regulates the development of root structures through polar auxin transport and promotes the growth of *A. thaliana* seedlings [22]. The endophytic bacterium *Bacillus subtilis* NUU4 of *Cicer arietinum* shows significant resistance to salinity and root rot 13 [23]. Endophytes can secrete and accumulate substances in the roots of plants, promote growth and development, increase stress and disease resistance, and enhance medicinal components. For example, *Bacillus altitudinis* KX230132.1, an effective activator, can increase the concentration of ginsenosides in the precious medicinal plant *Panax ginseng* [24]. The endophytic *Pseudomonas* increased the accumulation of sesquiterpenoids in *Atractylodes lancea* [25]. Therefore, the study of endophytes will help to further improve the medicinal value of plants.

Medicinal plants are often famous for their rich metabolites, and the content of important metabolites is also an important factor to consider when evaluating the quality of traditional Chinese medicine [26]. Metabolome analysis can evaluate and identify all metabolites in organisms under specific conditions, and has been widely used in the research of plant metabolites in recent years [27]. Flavonoids play an important role in improving crop tolerance to biotic and abiotic stresses [28]. Zhang et al. revealed the metabolic and functional roles of flavonoids in light-sensitive tea [29]. Pinasseau et al. analyzed the relationship between different polyphenol metabolites and related synthetic pathways through metabolome analysis, and established patterns of polyphenol responses to drought [30]. Through combined metabolome and transcriptome analyses, Yuan et al. revealed the correlation between genes and metabolites related to flavonoid biosynthesis in *Dendrobium moniliforme* as well as new regulatory mechanisms related to flavonoid biosynthesis [31]. Therefore, metabolome analysis is an effective tool to elucidate the potential medicinal value of medicinal plants.

At present, complex biological problems can no longer be clearly explained by a single omics approach, and the joint analyses of multiple omics is particularly important. In this study, we comprehensively analyzed the root microbiome and non-targeted metabolome of the medicinal cultivar ‘Hangbaishao’ (HS) and the ornamental cultivar ‘Zifengyu’ (ZFY) and compared the microbial composition and metabolites in the roots of two *P. lactiflora* cultivars. By comparing and analyzing medicinal components, such as flavonoids, in the roots of medicinal and ornamental cultivars of *P. lactiflora*, the potential medicinal value of ornamental varieties was discussed, and a new approach was provided for the realization of the “dual use of medicine and appreciation”.

## 2. Results

### 2.1. The Abundance and Diversity of Endophytic Microorganisms in HS and ZFY Roots

**Figure 1:**
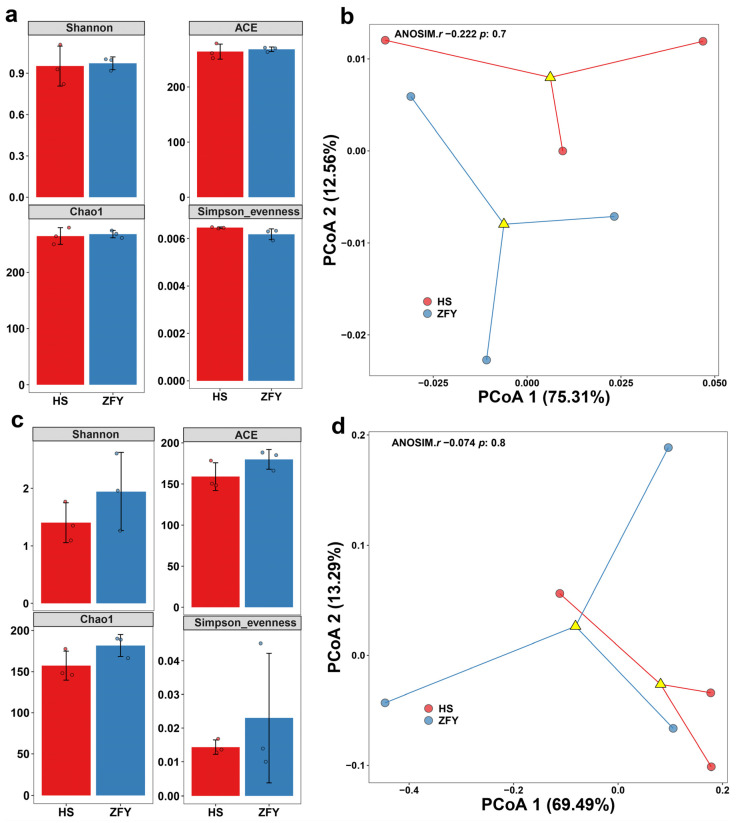
Alpha and beta analyses of endophytic microorganisms in HS and ZFY roots. Shannon diversity, ACE estimator, Chao1 estimator, and Simpson index of the bacterial communities (**a**) and fungal communities (**b**). Principal coordinate analysis (PCoA) of the bacterial communities (**c**) and fungal communities (**d**).

Simpson index, Chao1 estimator, ACE estimator, and Shannon diversity index were calculated to compare endophyte abundance and diversity in HS and ZFY (Figure 1). The results showed that the diversity and abundance of bacteria in HS and ZFY were similar (Figure 1a). However, among fungi, the Chao1 and ACE estimators of HS were much lower than those of ZFY, indicating that the abundance of fungi in HS was much lower than that in ZFY. The Simpson and Shannon diversity indexes showed that the diversity of fungi in ZFY was higher than that in HS (Figure 1c). In addition, principal coordinate analysis (PCoA) was conducted to isolate all samples containing bacterial and fungal communities. In the bacterial PCoA, PCoA1 and PCoA2 explained 75.31% and 12.56% of the total variance, respectively (Figure 1b). In fungal PCoA, PCoA1 accounted for 69.49% of the total variance and PCoA2 accounted for 13.29% (Figure 1d).

### 2.2. Species Composition and Abundance of Endophytic Microbial Communities of HS and ZFY

**Figure 2:**
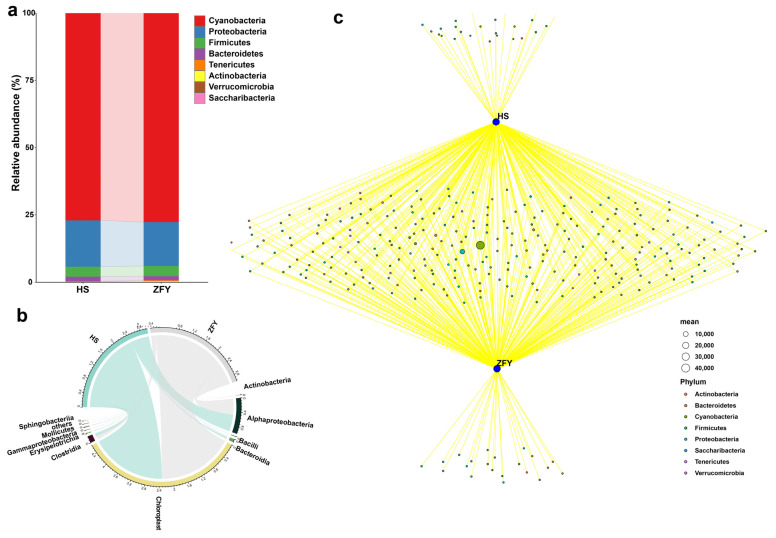
Composition of the endophytic bacterial communities in HS and ZFY. (**a**) Relative abundance of phyla in the bacterial communities. (**b**) Composition of class of bacterial communities. (**c**) Network Venn diagram showing positive associations between different species (HS and ZFY) and significantly associated bacterial phyla.

The species and relative abundance of bacteria and fungi between HS and ZFY were similar, but there were also some differences. At the phylum level, the composition of endophytic bacteria in HS and ZFY was similar. *Cyanobacteria* had the highest relative abundance, followed by *Proteobacteria*, *Firmicutes*, and *Bacteroidetes*, and the relative abundances of *Tenericutes* and *Actinobacteria* were small (Figure 2a). The commonality and specificity of HS and ZFY endophytic bacteria were further explored by drawing a Venn diagram to evaluate the contribution of different endophyte populations to the entire microbial community structure. Among all the bacterial communities, the two bacteria with the highest abundance shared by HS and ZFY belonged to the phyla *Cyanobacteria* and *Proteobacteria*, respectively. In addition, at the phylum level, one bacterium of *Actinobacteria*, four *Cyanobacteria*, nine *Firmicutes*, and seven *Proteobacteria* were specific to HS. Two bacteria of *Actinobacteria*, two *Bacteroidetes*, three *Cyanobacteria*, one *Firmicutes* and thirteen *Proteobacteria* were unique to ZFY (Figure 2c). At the class level, *Chloroplast* and *Alphaproteobacteria* were the most abundant endophytic bacteria in both HS and ZFY (Figure 2b).

**Figure 3:**
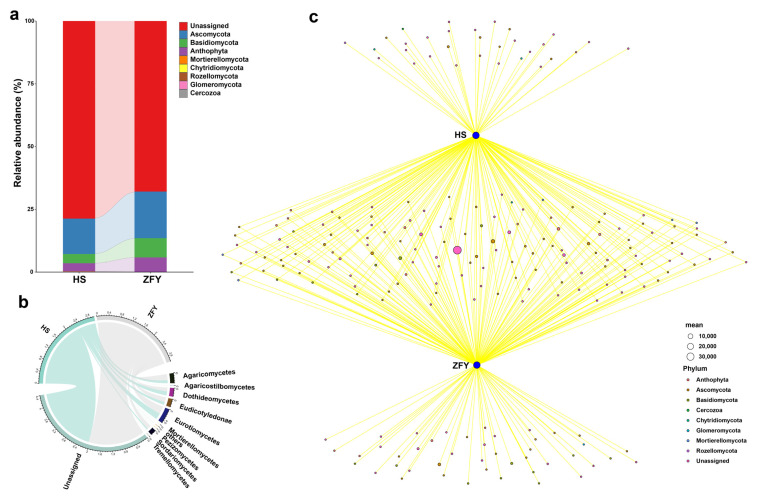
Composition of the endophytic fungal communities in HS and ZFY. (**a**) Relative abundance of fungal communities. (**b**) Composition fungal community classes. (**c**) Network Venn diagram showing positive associations between different species (HS and ZFY) and a significantly associated fungal phylum.

The relative abundances of endophytic fungi in HS and ZFY were significantly different at the phylum level. Although the three phyla with the highest relative abundance in HS and ZFY were *Ascomycota*, *Basidiomycota*, and *Anthophyta*, their relative abundances in ZFY were significantly lower than those in HS (Figure 3a). The network Wayne diagram showed that among the fungi peculiar to HS, two belonged to the phylum *Anthophyta*, six to *Ascomycota*, one to *Chytridiomycota*, and one to *Rozellomycota*. Among the unique fungi of ZFY, four belonged to the phylum *Anthophyta*, thirteen belonged to *Ascomycota*, nine belonged to *Basidiomycota*, and one belonged to *Glomeromycota* (Figure 3c). At the class level, the highest abundances of endophytic fungi in HS and ZFY were observed in *Eurotiomycetes* (Figure 3b).

### 2.3. Widely-Targeted Metabolomics Analysis of HS and ZFY

**Figure 4:**
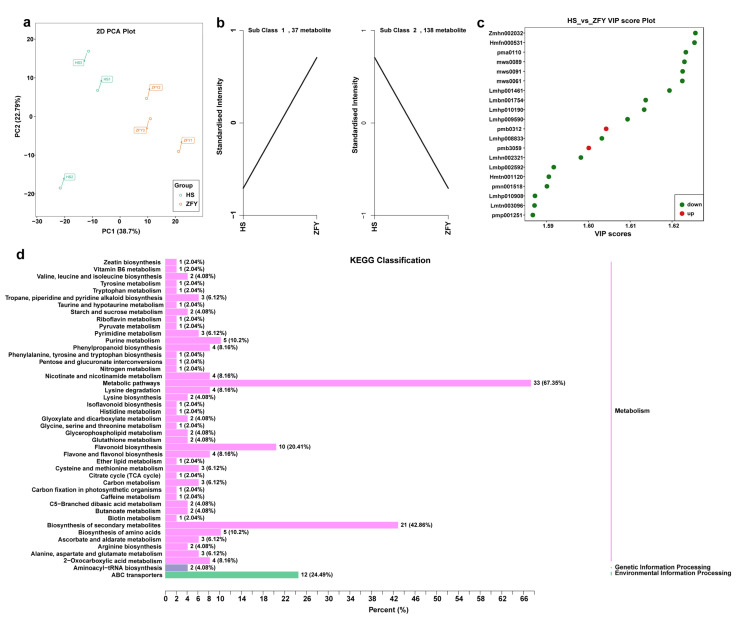
Metabolome analysis of HS and ZFY. (**a**) Two-dimensional principal component analysis (2D PCA). PC1 denotes the first principal component and PC2 denotes the second principal component. This percentage indicates the explanation rate of the principal component of the dataset. (**b**) K-means analysis showing trends in the relative content of metabolites in different subgroups. The mean values of the relative contents of differential metabolites in each group were standardized using z-score and subjected to K-means clustering analysis. The horizontal coordinate indicates the name of the sample, the vertical coordinate indicates the relative content of standardized metabolites, the sub-class represents the number of metabolite classes with the same trend of change. (**c**) VIP score plot showing the results of differentially expressed metabolites with the top ranked VIP values in the OPLS-DA model for each group comparison. Horizontal coordinates indicate VIP values, vertical coordinates indicate differential metabolites, red represents upregulated differentially expressed metabolites and green represents downregulated differentially expressed metabolites. (**d**) KEGG pathway analysis of differential metabolites. The vertical coordinate is the name of the KEGG metabolic pathway and the horizontal coordinate is the number of metabolites annotated to that pathway and their number as a percentage of the total number of annotated metabolites.

The extensively targeted metabolomes of HS and ZFY roots were analyzed using LC-MS to study metabolic differences. The PCA results showed that all samples were successfully separated into two clusters, indicating good reproducibility and low variability (Figure 4a). A total of 681 differential metabolites were identified, including 74 flavonoids, 112 phenolic acids, 114 lipids, 76 amino acids and their derivatives, 48 tannins, 40 nucleotides and their derivatives, 60 organic acids, 36 alkaloids, 24 terpenoids, 14 lignans and coumarins, and 83 others. To study the changing trend of the relative content of metabolites in HS and ZFY, K-means analysis was carried out. The results showed that among the 175 differential metabolites detected, 37 were upregulated and 138 were downregulated (Figure 4b). The top 20 differentially expressed metabolites with VIP values in the OPLS-DA model were selected for the analysis. The downregulated metabolites included five lipids, four flavonoids, four phenolic acids, one amino acid and derivatives, one ligan and coumarins, one vitamin, one tannin, and one organic acid, whereas the upregulated metabolites included one alkaloid and one organic acid (Figure 4c). In addition, KEGG was annotated for significantly different metabolites and classified according to pathway type. The results showed that the differential metabolites were mainly concentrated in metabolic pathways, biosynthesis of secondary metals, and flavonoid biosynthesis. The above results show that there were significant differences in the metabolites between HS and ZFY, among which the main differential metabolites were lipids, flavonoids, and phenolic acids (Figure 4d). The above results illustrate that the metabolites were significantly different between HS and ZFY, among which the main differential metabolites were lipids, flavonoids, and phenolic acids.

### 2.4. Joint Analyses of Metabolome and Microbiome

**Figure 5:**
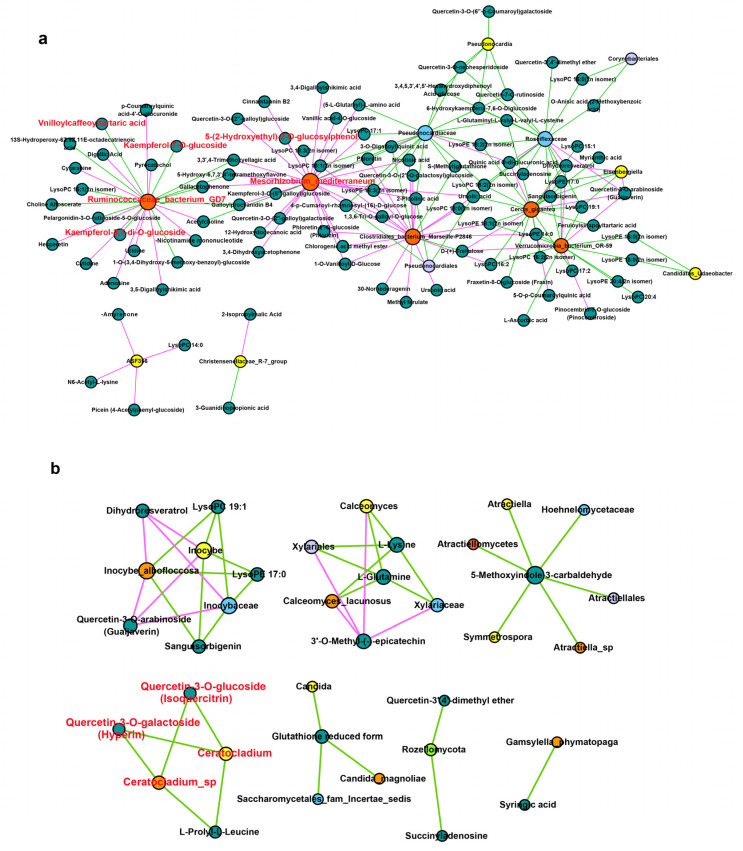
Correlation analysis of metabolome and microbiome. (**a**) Correlation analysis between metabolites and bacteria, *p* < 0.05. (**b**) Correlation analysis between metabolites and fungi, *p* < 0.01. Metabolites are shown in a green circle, yellow circle shows genus, lavender circle shows order, orange-red circle shows class, blue circle shows family, orange circle shows species, and light green circle shows phylum. The positive correlation is shown by the red line, the negative correlation is shown by the green line, and the size of the circle indicates the size of connectivity.

To further understand the regulatory relationship between endophytes and secondary metabolites and their effects on HS and ZFY metabolism, multi-omics analysis was used to analyze the correlation between the microbiome and the metabolome. Correlation analysis revealed a potential interaction between endophytes and secondary metabolites in *P. lactiflora* roots. To explore the potential medicinal value of *P. lactiflora*, we focused on the relevance of medicinal ingredients, such as flavonoids and phenolic acids, to endophytes. In bacteria, two flavonoid differential metabolites Kaempferol-7-O-glucoside, Kaempferol-3,7-di-O-glucoside and 1 phenolic acid differential metabolite, Vnilloylcaffeoyltartaric acid, were significantly correlated with *Ruminococcaceae bacterium* GD7 (Figure 5a). The phenolic acid 5-(2-Hydroxyethyl)-2-O-glucosylphenol was significantly correlated with *Mesorhizobium mediterranenum*. In fungi, differential flavonoid metabolites Quercetin-3-O-glucoside (Isoquercitrin) and Quercetin-3-O-galactoside (Hyperin) were significantly correlated with *Ceratocladium* sp. and *Ceratocladium.* It can be seen that the accumulation of secondary metabolites was significantly correlated with the endophytes, and the endophytes in the root of *P. lactiflora* can promote the accumulation of secondary metabolites (Figure 5b).

## 3. Discussion

Microbes growing in plant tissues may cause various changes in the metabolome by inducing plant biosynthetic pathways [32]. In our study, there were differences in the composition of root metabolites and endophytes between HS and ZFY. Although the diversity and abundance of bacteria were not significantly different between HS and ZFY, the diversity and abundance of endophytic fungi in the ornamental cultivar ZFY were much higher than those in the medicinal cultivar HS. In the analysis of differential metabolites, the phenolic acid and flavonoids contents in the ornamental cultivar ZFY were significantly higher than those in the medicinal cultivar HS. Therefore, we speculated that the difference in endophytic flora between HS and ZFY might lead to differences in phenolic acid and flavonoid metabolites.

Phenolic acids and flavonoids are the main substances in the extracts of endophytes and host plants and have high medicinal value [33]. Phenolic acids have antibacterial, anti-cancer, anti-inflammatory, and antioxidant properties [34,35]. Phenolic acid compounds in *P. lactiflora* include gallic acid, catechin, and other important medicinal components [36,37]. Flavonoids have been proven to have antioxidant, anti-inflammatory, anti-allergic, anti-cancer, and anti-diabetic activities [38,39,40]. As an ornamental cultivar, ZFY has much higher phenolic acids and flavonoids in the roots than the medicinal cultivar HS, and the composition of flavonoids in ZFY is significantly different from that in HS in our previous metabolome. The flavonoids in HS were mainly flavonoids and flavonol glycosides, whereas those in ZFY were mainly dihydroflavonoids and dihydroflavonols. This shows that ZFY not only has a high ornamental value, but also has potential medicinal value. Several studies have confirmed the medicinal value of ornamental plants. For example, phenols and flavonoids extracted from the famous ornamental plant *Rosa chinensis* have strong antioxidant activity [41]. The ornamental plant *Narcissus* was found to contain a large amount of alkaloids, which can be used to treat Alzheimer’s disease [42]. Hanieh et al. reviewed studies on the therapeutic potential and pharmacological activity of ornamental *Chrysanthemum* and confirmed its potential for medicinal development [43].

Currently, research on new drugs focuses on medicinal compounds related to endophytes [44]. The endophytic fungus *Gilmaniella* sp. of *Atractylodes lancea* can induce the production of jasmonic acid to accumulate medicinal sesquiterpenoids [45]. The secondary metabolites produced by the endophytic fungus *Papulaspora immersa* of *Smallanthus sonchifolius* showed high anti-tumor activity [46]. Endophytic fungi in *Compositae*, such as *Fusarium*, *Plectosphaerella*, *Stemphyum*, and *Septoria*, can produce anti-cancer enzymes for the treatment of acute lymphoblastic leukemia [47]. It can be seen that the endophytes of medicinal plants interact with plants and act on secondary metabolites together. To explore the flora related to the accumulation of medicinal metabolites, such as phenolic acids and flavonoids, as in ZFY, a correlation analysis was performed using multi-omics analysis technology. Through the analysis of the correlation results, we focused on the bacteria *R. bacterium* GD7, which is significantly related to differential metabolites of flavonoids and phenolic acids, and the bacteria *M. mediterraneum*, which is related to phenolic acids. In addition, we focused on the fungus *Ceratocladium* sp., which is significantly related to differential flavonoid metabolites. Therefore, we tentatively speculate that bacteria *R. bacterium* GD7, *M. mediterranenum* and fungi *Ceratocladium* sp. may be related to regulating the synthesis of phenolic acids and flavonoids metabolites in the root of *P. lactiflora*. However, in our study, the bacteria *M. mediterraneum* and the fungus *Ceratocladium* sp. were only found in HS, whereas phenolic acid and flavonoid metabolites produced by HS were much lower than those produced by ZFY. The abundance of *R. bacterium* GD7 in ZFY was much higher than in HS, which was significantly associated with the production of phenolic acid and flavonoid metabolites. In addition, previous studies have found that the endophyte *Ruminococcaceae* in *Ginkgo biloba* is significantly positively correlated with the flavonoid accumulation of flavonoids [48]. Therefore, it appears that *R. bacterium* GD7 is the key bacterium responsible for the accumulation of phenolic acids and flavonoids in ZFY.

*P. lactiflora* is an important plant for both medicinal and ornamental purposes, but most species are used for ornamental purposes and are neglected for medicinal purposes. The root endophytic bacteria and differential metabolites were compared between the medicinal variety HS and ornamental variety ZFY, confirming the medicinal value of the ornamental variety ZFY. Through joint analyses of the microbiome and metabolome, the key endophytes related to the accumulation of pharmaceutical ingredients were identified, which is helpful for further exploring the potential pharmaceutical value of the roots of *P. lactiflora* in the future.

## 4. Materials and Methods

### 4.1. Plant Material and Sample Collection

The study area was located at the Horticulture and Plant Protection College of Yangzhou University, Jiangsu province, China (32°30′ N, 119°25′ E). A subtropical monsoon climate prevails in this region, with a mean annual precipitation of 991 mm and a mean annual temperature of 15.2 °C. Wild plants were managed according to the standard cultivation practices in the natural environment. At the end of August 2020, the three-year-old ornamental cultivar ‘Zifengyu’ (ZFY) and medicinal cultivar ‘Hangbaishao’ (HS) of *P. lactiflora* were selected for this study. Three different individuals from each cultivar were collected in situ and were uniform and separated by at least 5 m.

The entire root system of each plant was sampled and treated separately. The soil on the root surface was carefully removed and rinsed with tap water. Roots with a diameter ≥ 2 mm were selected and surface-disinfected by shaking for 4 min in 2% NaClO. After sterilization, the root systems were rinsed 5 times with sterile Milli-Q water to remove NaClO residue.

### 4.2. Widely-Targeted Metabolomics Profiling

To explore the differences in metabolite composition between the roots of HS and ZFY, Metware Biotechnology Ltd. (Wuhan, China) was contacted and metabolome analysis was conducted on samples with three biological duplicates for each cultivar. The roots of HS and ZFY were freeze-dried and crushed in a vacuum freeze dryer (ScientZ-100F) and a mixing mill (MM400, Retsch), respectively. A solution of 1.2 mL 70% methanol was then used to dissolve 100 mg of the lyophilized powder, which was then extracted by shaking and stored overnight at 4 °C. After centrifugation and filtration (SCAA-104, 0.22 μm aperture; ANPEL, Shanghai), the extracts were analyzed using an UPLC-ESI-UPLC-MS/MS system (UPLC, NexeraX2; MS, biological system 4500QTRAP). Metabolic data were analyzed using program Analyst 1.6.3, and the differential metabolites between the two samples were identified by orthogonal projections to latent structures-discriminant analysis (OPLS-DA). Based on the OPLS-DA results, the derived variable importance in projection (VIP) of the OPLS-DA model was used for multivariate analysis, and differential accumulative metabolites (DAM) were preliminarily screened. We used |log_2_ (fold change)| ≥ 1 and VIP ≥ 1 for the DAM in the next step of our study.

### 4.3. DNA Extraction, Amplification, and Sequencing

Three replicates of each sample were prepared to extract the DNA from the endosphere. Total genomic DNA was extracted using the cetyltrimethylammonium bromide (CTAB) technique [49]. DNA content and purity were evaluated using 1% agarose gel electrophoresis. The DNA was diluted to 1 ng·μL^−1^ with sterile water, depending on the concentration. The primers 799F (AACMGGATTAGATACCCKG) and 1193R (ACGTCATCCCCACCTTCC) were used to generate bacterial libraries by amplifying the V5–V7 region of the 16S rRNA gene using the unique 6-nt barcode at the 5′ end of the forward primer. Fungal libraries were constructed similarly to bacterial libraries, except that they were amplified using ITS1F (CTTGGTCATTTAGAGGAAGTAA) and ITS1R (GCTGCGTTCTTCATCGATGC) for the ITS1b region. PCR was performed using the Phusion^®^ High-Fidelity PCR Master Mix (New England Biolabs, Ipswich, MA, USA). Subsequently, the PCR products were mixed with an equal volume of 1 × loading buffer (containing SYB green) and detected using 2% agarose gel electrophoresis. The PCR products were mixed at an equidensity ratio. The PCR products were purified using a GeneJETTM Gel Extraction Kit (Thermo Scientific, Waltham, MA, USA). Following the manufacturer’s recommendations, sequencing libraries were generated using the Truseq^®^ DNA PCR-Free Sample Preparation Kit (Illumina, San Diego, CA, USA), and index codes were added. The Agilent Bioanalyzer 2100 system and Qubit^@^ 2.0 Fluorometer (Thermo Scientific, Waltham, MA, USA) were used to assess the quality of the library. The Illumina MiSeq PE300 platform was used to sequence the libraries and generate paired reads.

### 4.4. Sequence Analysis

Raw data from the 16S V5–V7 bacterial region and the ITS1b fungal region were processed using QIIME for quality-controlled processing (V1.9.1, http://qiime.org/scripts/splitlibrariesfastq.html (accessed on 9 February 2022)) [50] and FLASH for paired reads (V1.2.7, http://ccb.jhu.edu/software/FLASH/ (accessed on 10 February 2022)) [51]. For bacteria and fungi, annotation was performed by matching the Silva sequences with the UCHIME algorithm and the Unite database (ITS: http://unite.ut.ee/ (accessed on 11 February 2022)) (UCHIME, http://www.drive5.com/usearch/manual/uchimealgo.html (accessed on 11 February 2022)) [52,53] UCHIME Algorithm (UCHIME Algorithm) [54]. Uparse was used to assign sequences with 97% similarity to the same OTUs (Uparse v7.0.1001; http://drive5.com/uparse/ (accessed on 12 February 2022)) [55].

### 4.5. Joint Analyses of Metabolome and Microbiome

To explore the correlation between the differential endophytes and their metabolites, we plotted a significant correlation analysis network. The correlation analysis of the detected differential endophytes and differential metabolites was performed and the Spearman correlation coefficient was calculated. Differential endophytes and metabolites with a correlation greater than 0.7 and significant correlation of *p* < 0.05 in bacteria and *p* < 0.01 in fungi were selected, and the correlation network was drawn using Cytoscape software [56].

### 4.6. Statistical Analysis

All analyses and figures were constructed using R software (Version 3.6.1; https://www.r-project.org/ (accessed on 13 February 2022)). We used the ‘dplyr’ software for data cleaning. Packages ‘vegan’, ‘phyloseq’, and ‘ggplot2’ were used to calculate the alpha diversity indexes such as Shannon, Pielou, Chao1, and ACE [57,58,59]. Beta-diversity was calculated by the package ‘phyloseq’ and visualized by the package ‘ggplot2’. A histogram of the top 10 relative abundances at the phylum and genus levels was also generated using the ‘ggplot2’ package. Finally, the ‘ggClusterNet’ package was used to create correlation network diagrams of bacterial communities (https://github.com/taowenmicro/ggClusterNet (accessed on 14 February 2022)).

## Data Availability

The NCBI Sequence Read archives contain raw Illumina sequence data for bacterial 16S rRNA and fungal ITS sequences, with accession number PRJNA665305.
